# Intravescical prostatic protrusion is a predictor of alpha blockers response: results from an observational study

**DOI:** 10.1186/s12894-018-0320-0

**Published:** 2018-02-02

**Authors:** L. Topazio, C. Perugia, C. De Nunzio, G. Gaziev, V. Iacovelli, D. Bianchi, G. Vespasiani, E. Finazzi Agrò

**Affiliations:** 1 School of Specialization in Urology, University “Tor Vergata”, Rome, Italy; 2 Department of Urology, Sant’Andrea Hospital, University “La Sapienza”, Rome, Italy; 3 Department of Experimental Medicine and Surgery, University “Tor Vergata”, Rome, Italy

**Keywords:** LUTS, BPH, Alpha-blockers, Intravesical prostatic protrusion

## Abstract

**Background:**

To investigate the efficacy of tamsulosin in patients with lower urinary tract symptoms (LUTS) and benign prostatic enlargement (BPE) with intravesical prostatic protrusion (IPP). Ultrasound measurement of the IPP has been previously described as an effective instrument for the evaluation of benign prostatic obstruction (BPO) and could help in clarifying the role of alpha-blockers in patients with (BPE).

**Methods:**

Patients with BPE and LUTS were enrolled in this observational study. Intravesical prostatic protrusion was graded as grade 1 (< 5 ml), 2 (5 < IPP < 10 ml) and 3 (> 10 ml). Patients were treated with tamsulosin for twelve weeks. Evaluation was performed before and at the end of treatment by means of International Prostate Symptom Score (IPSS) and uroflowmetry. Patients were considered responders if a reduction of IPSS > 3 points was reported.

**Results:**

One hundred forty-two patients were enrolled. Twelve patients were excluded because of incomplete data. Fifty patients showed an IPP grade 1 (group A), 52 a grade 2 (group B) and 28 a grade 3 (group C). Treatment success was obtained in 82%, 38,5% and 7,1% of patients respectively; these differences (group A vs B-C and group B vs C) were highly significant. The odd ratio to obtain a treatment success was of 59 and 8.1 in group A and group B respectively, in comparison to group C. After a multivariate regression, the relationship between IPP grade and treatment success remained significant. Improvement of uroflowmetry parameters has been reported in all the groups especially in patients with a low grade IPP (*p* value = 0,016 group A vs group B; *p* value = 0,005 group A vs group C). Prostate volume seems not to influence this relationship.

**Conclusions:**

Intravesical prostatic protrusion has found to be significantly and inversely correlated with treatment success in patients with LUTS and BPE under alpha-blockers therapy. Alpha blockers odd ratio of success is 59 times higher in patients with a low grade IPP in comparison to patients with a high grade.

## Background

Benign prostatic hyperplasia (BPH) is found in over half of 60-year-old men and in almost all of 80-year-old men [1] and is the most frequent cause of bladder outlet obstruction (BOO) in males over the age of 50 who apply with lower urinary tract symptoms [2]. In this case, the term Benign Prostatic Obstruction (BPO) is currently used [3]. In BPO patients, medical therapy is the most commonly used [3] and provides relief in symptoms and alteration in disease progression [4]. However, long-term dropout rates reach 30–43% [5] and not all patients benefit from the treatment. Therefore, it would be beneficial to identify patients that will not respond to medical treatment.

The evaluation of BPO is an important factor that can reflect the severity of disease and can aid in measuring the outcome of the treatment. Nowadays the standard practice investigation for BPO patients is composed of uroflowmetry and ultrasound (US) evaluation of the post-void residual urine (PVR) [3]. Unfortunately, such investigations have less possibility to clearly identify the degree of BOO in men affected by Benign prostatic enlargement (BPE) with respect to pressure-flow study (PFS) during invasive urodynamic investigation (UD) [6]. However, UD test before surgical BPO treatment is not always indicated in international guidelines because of the invasiveness and high costs of the method [3, 7].

In the last decade, several authors have tried to identify novel and less invasive parameters that could help the physician in evaluating the degree of BPO, thus predicting a treatment response. The most promising among them are bladder/detrusor wall thickness [8, 9], ultrasound-estimated bladder weight [10], non-invasive pressure-flow testing [11], prostatic urethral angle [12] and US measurement of the intravesical prostatic protrusion (IPP) [11, 13, 14].

US measurement of IPP was first described by Chia et al. in 2003 to correlate well with BPO (presence and severity) on urodynamic testing, with a PPV of 94% and a NPV of 79% [13]. The clinical significance of IPP can be explained by the fact that protrusion of the median lobe of the prostate into the bladder can cause a “ball valve” type of benign prostatic obstruction with incomplete opening and disruption of the funneling effect of the bladder neck [13, 15].

Further studies on this topic have shown that IPP may correlate with prostate volume, detrusor overactivity (DO), bladder compliance, detrusor pressure at maximum urinary flow, BOO index and PVR, and negatively correlates with Qmax [16]. Moreover, IPP also seems to predict successfully the outcome of a trial without catheter (TWOC) after acute urinary retention [17] and the success rate of TURP [18]. To date, however, few data have been reported in terms of the association between IPP and clinical outcomes in patients undergoing medical therapy. Studies investigating the relationship between IPP and alpha-blockers therapy outcomes [19, 20] have shown that it may be correlated to reduced efficacy of alpha blockers in patients with IPP and mild/moderate (< 40 ml) prostate volume (PV) [19, 20]. However, to our knowledge, no data are available on patients with PV ≥ 40 ml.

Aim of this study was to investigate the efficacy of an alpha-blocker (Tamsulosin) in patients with lower urinary tract symptoms (LUTS) and BPE with or without IPP.

## Methods

This is an observational prospective study performed from January to December 2015 in the outpatient clinic of Tor Vergata University Hospital in Rome and reported following the STROBE statement.

We enrolled male patients between fifty and seventy-five years of age, affected by BPE defined as trans-rectal ultrasound (TRUS) estimated PV ≥ 30 ml, in whom tamsulosin had been prescribed for LUTS.

Exclusion criteria were:

- Prior urologic surgery;
- Patients affected by a urologic neoplasia, bladder calculus or any type of neurological abnormality;
- Prior treatment with alpha blockers and 5alpha reductase inhibitors;
- Absence of intravescical prostatic protusion.

All patients enrolled underwent a baseline evaluation by means of medical history, administration of the International Prostate Symptom Score and Quality of Life (IPSS/QoL) questionnaire, trans-rectal ultrasound of the prostate and uroflowmetry. All TRUS were performed by the same physician and at the standard bladder filling of 150 ml. Trans rectal ultrasound was performed in the midsagittal plane and IPP along with the prostatic volume were measured. IPP was identified according to the classification system used by Nose et al [21] and was defined by the distance from the tip of the prostate’s protrusion into the vesical lumen to the bladder neck measured in millimetres. IPP estimated by TRUS was then graded as Grade 1 (if it was inferior to 5 mm), Grade 2 (if it was comprised between 5 and 10 mm) and Grade 3 (if it was superior to 10 mm). PV measurement was obtained during TRUS. All uroflowmetry were performed at the standard bladder filling of 250–300 ml as recommended by the guidelines for good urodynamic practices [22].

All patients enrolled were then treated with Tamsulosin (0,4 mg/day) for twelve weeks and re-evaluated after treatment by means of International Prostate Symptom Score and Quality of Life (IPSS/QoL) and uroflowmetry. Patients were considered responders (treatment success) if showing a reduction of IPSS > 3 points.

### Statistical analysis

All data were classified in an Excel Database. All analyses were performed by means of the software STATA 13.0. Univariate logistic regression was used to evaluate relationships between each parameter (IPP grade, PV, IPSS, Qmax, PSA) and treatment success. A paired t test was used to evaluate change in time in uroflow parameters in each group separately. One way anova was used to compared these changes between the three groups. Bonferroni correction was applied in post hoc comparison. Univariable logistic regression was used to evaluate relationships between IPP grade and the treatment success. Odd Ratio (OR) and relative 95% confidence Interval (CI 95%) were reported. A stepwise logistic regression was applied considering as independent factor IPP grade, age PSA, PV and baseline value of Qmax IPSS and RPM. Adjusted OR (ORadj) and relative 95% confidence Interval (CI 95%) were reported. A *p* value < 0.05 was considered statistically significant.

## Results

One hundred forty-two patients were enrolled. Twelve patients were excluded because of incomplete data. Of the remaining 130 patients, 50 (38.5%) showed an IPP grade 1 (group A), 52 (40%) an IPP grade 2 (group B) and 28 (21.5%) an IPP grade 3 (group C). Baseline features of patients are showed in Table 1. Treatment success, defined as post-treatment IPSS score reduction > 3 points, was obtained in 82%, 38,5% and 7,1% of patients respectively. The odd ratio to obtain a treatment success was of 59 (CI 95% 11.8–296) and 8.1 (CI95% 1.7–38) in group A and group B respectively, in comparison to group C (Table 2). Moreover, there is a positive improvement of uroflow parameters in each group (Table 3) with a better improvement after treatment in patients with a low grade IPP with respect to patients with a higher grade IPP (*p* value = 0,016 Group A vs Group B; *p* value = 0,005 Group A vs Group C).

**Table 1:** Baseline features of Patients

	Age, mean (DS)	Prostate volume, mean (DS)	Estimated IPP, mean (DS)	Pre-treatment Qmax, mean (DS)	Pre-treatment IPSS, mean (DS)	Pre-treatment PSA, mean (DS)
Group A	62 (8.9)	45.5 (16.9)	2.7 (0.8)	10.5 (2.9)	17.7 (3.9)	3.5 (2.5)
Group B	64 (9)	53.2 (21.2)	6.5 (1.3)	9.3 (1.5)	18 (4.1)	2 (1.4)
Group C	66 (8.6)	54.6 (13.1)	11.4 (1.1)	8.8 (2.3)	22.2 (5.1)	3.1 (1.7)

*IPP* Intravesical prostatic protrusion, *Qmax* Maximum flow at uroflowmetry, *IPSS* International prostatic symptoms score

**Table 2:** Relationship between IPP grade and treatment success

	Responders	Not responders	OR	CI 95%	*p*
Group A	41 (65.1%)	9 (13.4%)	59	11.8–296	< 0.001
Group B	20 (31.7%)	32 (47.8%)	8.1	1.7–38	0.008
Group C	2 (3.2%)	26 (38.8%)	1		

*OR* Odd ratio, *CI* Confidence interval

**Table 3:** Pre post-treatment Qmax differences

	Pre-treatment Qmax, mean (SD)	Post-treatment Qmax, mean (SD)	Pre post-treatment Qmax differences, mean (SD)	pValue
Group A	10.5 (2.9)	14.1 (3.2)	3.6 (1.4)	< 0.001
Group B	9.3 (1.5)	11.7 (2.7)	2.4 (2.7)	< 0.001
Group C	8.8 (2.3)	10.8 (2.7)	2.0 (2.2)	< 0.001

*Qmax* Maximum flow at uroflowmetry

After multivariate regression, the relationship between IPP grade and treatment success remained significant. Interestingly the multivariate regression shows that PV seems not influence this relationship and is not included in the final model (Table 4).

**Table 4:** Multivariate regression on treatment success

	ORadj	CI 95%		*P* value
IPP grade				
Grade 1	84.83	14.31	502.93	< 0.001
Grade 2	8.70	1.70	44.51	0.009
Grade 3	1			
Age	.95	.90	1.00	0.071
PSA	.74	.58	.94	0.014
PVR pre	.99	.97	.99	0.037

Stepwise regression. Initial model: IPP grade, PSA, PV, Qmax baseline, IPSS baseline, RPM baseline

## Discussion

IPP is a promising parameter, first described by Chia in 200, 313, that has shown a good correlation with the presence and severity of BPO on urodynamic testing. Further studies have found a strong correlation between IPP and DO, bladder compliance, detrusor pressure at maximum urinary flow, terminal dribbling, BOO index and PVR while a negative correlation was found between IPP and Qmax and/or alpha-blockers efficacy [16, 19, 20, 23]. Moreover a well-designed study from Luo GC et al. has shown that the presence of middle lobe is more obstructive than those of lateral lobes and could better correlate with BOO grade [24]. Data coming from our study suggest that IPP is significantly and inversely correlated with treatment success in patients affected by BPE and with LUTS under alpha-blocker therapy. Alpha-blockers odd ratio of success is 59 times higher in patients with a low grade IPP in comparison to patients with a high grade IPP and 8 times higher with respect to patients with a moderate grade IPP. It is important to underline that the definition of success used in this study (a reduction of IPSS score > 3 points) is in line with previous and contemporary studies, considering this IPSS variation as clinically significant [25]. Interestingly even after multivariate regression with stepwise logistic regression IPP remains as independent predictive factors for alpha blockers treatment success.

Our data are similar to those in literature; Cumpanas et al. analyzed 183 patients with BPH (PV < 40 mL) treated with tamsulosin and found that approximately 40% of the patients in the high IPP group were treatment nonresponders and had significantly worse outcomes than patients in the low IPP group at 3 months [20]*;* even in a more recent paper Kalkanli et al. [26] showed that an increase in IPP was associated with a lower response level to medical treatment and indicated a significant negative correlation between IPP-Qmax and IPP-post treatmens IPSS. Similar data were also published by Hirayama et al [27] in Patients treated with Dutasteride 0.5 mg daily in which IPP was seen to be the strongest predictive factor for failure of medical therapy and conversion to surgical intervention with the optimal cutoff value of IPP of 8 mm. This value yielded a sensitivity of 91% and a specificity of 72%.

Interestingly our data show that PV seems not to influence the relationship between IPP and alpha-blockers success rate. This is, to our knowledge, one of the very first papers to investigate this relationship in Patients with PV higher than 40 ml and our results are similar to those already published by Wang et al. in 2015 [28] who showed a strong correlation between IPP and BPO and stated that IPP is superior to PV in predicting BPO in patients who present with LUTS.

Our result indicates that IPP helps to predict obstruction by BPH and therefore the progression of BPH (prostate adenoma) and response rate to alpha blockers therapy; therefore, IPP is useful in stratifying BPH patients with LUTS at initial evaluation, helping the urologist in deciding which patient could benefit from medical therapy and avoiding lots of unuseful prescriptions for further cost-effective management.

It is interesting to observe that also Qmax showed a larger improvement in low or moderate IPP grade patients in comparison to severe IPP grade patients (*p* value = 0,016 Group A-B vs Group C). Furthermore, patients with a higher IPP grade showed lower pre-treatment Qmax, in comparison with patients with 1–2 IPP grade (p value = 0,029 Group A-B vs Group C). This finding seems to suggest that the degree of IPP could correlate with the degree of BPO, but the lack of an invasive urodynamic evaluation (pressure/flow study) do not allow us to draw conclusions on that point.

The study has several limitations; no RCT, no sample size calculation; non-invasive UD data. Besides we are conscious that EAU Guidelines on “Non neurogenic male LUTS” [3] suggest a combination therapy for patients with PV > 40 ml and therefore that the vast majority of patients in our study did not receive a Guideline conform therapy but the aim of this study was not to evaluate the efficay of tamsulosin monotherapy in PV > 40 ml but to assess the efficacy of alpha-blockers in relationship to the IPP grade. Thus the results of this paper could reinforce the indication of a combination therapy in this patients’ population. This study provides furthermore the information that IPP seems to be a negative prognosctic factor for success of Tamsulosin, independently by the prostate volume.

The strengths of the study are a proper statistical methodology and the use of a clinically meaningful primary outcome measure as modifications in IPSS score.

## Conclusions

IPP seems significantly and inversely correlated with treatment success in patients with LUTS and BPE under alpha-blockers and may be considered a useful tool to discriminate patients in whom medical treatment has higher (low grade IPP) or lower (high grade IPP) probability of success.

## Abbreviations

BOO: Bladder outlet obstruction
BPE: Benign prostatic enlargement
BPH: Benign prostatic hyperplasia
BPO: Benign Prostatic Obstruction
CI: Confidence interval
DO: Detrusor overactivity
IPP: Intravesical prostatic protrusion
IPSS/QoL: International Prostate Symptom Score and Quality of Life
LUTS: Lower urinary tract symptoms
OR: Odd Ratio
PFS: Pressure-flow study
PV: Prostate volume
PVR: Post-void residual urine
TRUS: Trans-rectal ultrasound
TWOC: Trial without catheter
UD: Urodynamic investigation
US: Ultrasound
